# 4*β*-Hydroxycholesterol Signals From the Liver to Regulate Peripheral Cholesterol Transporters

**DOI:** 10.3389/fphar.2020.00361

**Published:** 2020-03-27

**Authors:** Tuire Salonurmi, Heba Nabil, Justiina Ronkainen, Tuulia Hyötyläinen, Heidi Hautajärvi, Markku J. Savolainen, Ari Tolonen, Matej Orešič, Päivi Känsäkoski, Jaana Rysä, Jukka Hakkola, Janne Hukkanen

**Affiliations:** ^1^Research Unit of Internal Medicine, University of Oulu, Oulu, Finland; ^2^Biocenter Oulu, Oulu, Finland; ^3^Research Unit of Biomedicine, University of Oulu, Oulu, Finland; ^4^Medical Research Center Oulu, Oulu University Hospital and University of Oulu, Oulu, Finland; ^5^Center for Life-Course Health Research, University of Oulu, Oulu, Finland; ^6^Department of Chemistry, Örebro University, Örebro, Sweden; ^7^Admescope Ltd., Oulu, Finland; ^8^School of Medical Sciences, Örebro University, Örebro, Sweden; ^9^Faculty of Health Sciences, School of Pharmacy, University of Eastern Finland, Kuopio, Finland

**Keywords:** 4*β*-hydroxycholesterol, ATP binding cassette transporter A1, ATP binding cassette transporter G1, inducible degrader of the LDL receptor, lectin-like oxidized LDL receptor-1, liver X receptor, pregnane X receptor

## Abstract

Activation of pregnane X receptor (PXR) elevates circulating 4*β*-hydroxycholesterol (4βHC), an agonist of liver X receptor (LXR). PXR may also regulate 25-hydroxycholesterol and 27-hydroxycholesterol. Our aim was to elucidate the roles of PXR and oxysterols in the regulation of cholesterol transporters. We measured oxysterols in serum of volunteers dosed with PXR agonist rifampicin 600 mg/day *versus* placebo for a week and analyzed the expression of cholesterol transporters in mononuclear cells. The effect of 4*β*HC on the transport of cholesterol and the expression of cholesterol transporters was studied in human primary monocyte-derived macrophages and foam cells *in vitro*. The expression of cholesterol transporters was measured also in rat tissues after dosing with a PXR agonist. The levels of 4*β*HC were elevated, while 25-hydroxycholesterol and 27-hydroxycholesterol remained unchanged in volunteers dosed with rifampicin. The expression of ATP binding cassette transporter A1 (ABCA1) was induced in human mononuclear cells *in vivo*. The influx of cholesterol was repressed by 4*β*HC, as was the expression of influx transporter lectin-like oxidized LDL receptor-1 *in vitro*. The cholesterol efflux and the expression of efflux transporters ABCA1 and ABCG1 were induced. The expression of inducible degrader of the LDL receptor was induced. In rats, PXR agonist increased circulating 4*β*HC and expression of LXR targets in peripheral tissues, especially ABCA1 and ABCG1 in heart. In conclusion, PXR activation-elevated 4*β*HC is a signaling molecule that represses cholesterol influx and induces efflux. The PXR-4*β*HC-LXR pathway could link the hepatic xenobiotic exposure and the regulation of cholesterol transport in peripheral tissues.

## Introduction

Like many other oxysterols, 4*β*-hydroxycholesterol (4*β*HC) is a ligand for liver X receptors (LXRs), the major regulators of lipid metabolism (Janowski et al., 1996; Lee and Tontonoz, 2015). Both LXRα (*NR1H3*) and LXRβ (*NR1H2*) are activated by 4*β*HC to a similar degree *in vitro* (Nury et al., 2013), but the role of 4*β*HC in the regulation of LXR targets is yet to be explored. LXR*α* is expressed in the liver, intestine, kidney, adipose tissue, adrenals, and macrophages, while LXR*β* is expressed ubiquitously (Lee and Tontonoz, 2015). The activation of LXR leads to upregulation of lipogenesis in the liver and induction of cholesterol efflux transporters such as ATP-binding cassette transporters A1 (ABCA1), ABCG1, and ABCG5/8, as well as repression of LDL receptor-mediated lipoprotein uptake in the liver and macrophages *via* inducible degrader of the LDL receptor (IDOL) (Lee and Tontonoz, 2015). The activation of LXR is generally considered to reduce atherosclerosis, while the induction of hepatic lipogenesis may lead to hepatosteatosis (Lee and Tontonoz, 2015).

The formation of 4*β*HC in the liver is mediated by cytochrome P450 (CYP) 3A4 and 3A5 enzymes, and serum/plasma 4*β*HC is considered as a novel endogenous marker of CYP3A4 and CYP3A5 activity (Diczfalusy et al., 2011). The range of serum 4*β*HC concentration varies more than 40-fold in subjects without CYP3A enzyme inducers and more than 100-fold when patients with enzyme inducers are included (Hole et al., 2017). The hepatic pregnane X receptor (PXR; *NR1I2*) and constitutively active receptor (CAR; *NR1I3*) are the main regulators of the induction of CYP3A enzymes (Zanger and Schwab, 2013). The administration of CYP3A inducers such as rifampicin or antiepileptics carbamazepine, phenytoin, and phenobarbital increases markedly the circulating 4*β*HC. The half-life of 4*β*HC is about 17 days in humans (Diczfalusy et al., 2011).

In addition to 4*β*HC, the production of 25-hydroxycholesterol (25HC) and 27-hydroxycholesterol (27HC) may be under the control of PXR. CYP3A4 may play a part in the hepatic production of 25HC (Honda et al., 2011), and PXR has been shown to activate the synthesis of 27HC by 27-hydroxylase (CYP27A1) in human intestinal cell models (Li et al., 2007). Also 25HC and 27HC are LXR agonists (Bovenga et al., 2015). Importantly, 25HC is a suppressor of interleukin-1 driven inflammation (Reboldi et al., 2014; Simon, 2014) as well as an antiviral factor (Liu et al., 2013), while 27HC promotes breast cancer metastasis by acting on immune myeloid cells (Baek et al., 2017).

As 4*β*HC is an agonist of LXR, we hypothesized that 4*β*HC, and possibly 25HC and 27HC, serve as signaling molecules that regulate peripheral cholesterol transporters. Thus, we set out to determine the role of 4*β*HC in human macrophage lipid transport. We also elucidated the effects of PXR activation on the expression of lipid transporters in peripheral tissues *in vivo*.

## Materials and Methods

### Subjects and Experimental Protocol

The protocol of the clinical study has been described in detail in the previous publication (Rysa et al., 2013). In short, healthy volunteers, aged 18–40 years, were recruited for the study. The study had a randomized, open, and placebo-controlled crossover design. Twelve individuals were administered 600 mg rifampicin (Rimapen; Orion Corporation, Espoo, Finland), an efficient PXR agonist, or placebo daily for a week with at least a 4-week washout period before the next treatment arm. At the end of each arm, blood samples were collected on the morning of the eighth day. The study was approved by the ethics committee of the Northern Ostrobothnia Hospital District (Oulu, Finland) and the Finnish Medicines Agency. Written, informed consent was obtained from each subject. The study procedures performed were in accordance with the ethical standards of the Helsinki Declaration. This trial was registered at ClinicalTrials.gov as NCT00985270.

### Analytical Methods

Total cholesterol, low-density lipoprotein cholesterol (LDL), high-density lipoprotein cholesterol (HDL), and triglycerides were assayed with an automatic chemical analyzer (Advia 1800; Siemens Healthcare Diagnostics GmbH, Eschborn, Germany) at the clinical laboratory of Oulu University Hospital (NordLab, Oulu, Finland) with a method validated for clinical use.

The analysis of 4*β*HC, 25HC, and 27HC was done at VTT Technical Research Centre of Finland Ltd. (Espoo, Finland) as described in (Hukkanen et al., 2015). Briefly, a 30 µl aliquot of serum was extracted with 400 µl chloroform:methanol (2:5, v:v) mixture, after addition of 10 µl of internal standard mixture (4*β*HC-D7, 25HCD6, desmosterol-D6, and 4(R/S),25-epoxycholesterol-D6; c = 0.25 mg/L) and after evaporation, the sample was derivatized with 40 µl of *N*-Methyl-*N*-(trimethylsilyl)trifluoroacetamide. The GC–MS system consisted of an Agilent 6890 gas chromatograph (Agilent Technologies, Santa Clara, CA, USA) combined with Agilent 5973 mass selective detector (MSD). Selective ion monitoring, using specific masses for each target analyte, was used in the detection. The calibration curves were constructed on the range of 1–200 ng/ml. The concentration of 25HC was below the limit of quantification in two subjects (one subject during rifampicin arm, another subject during placebo arm). In these cases, 25HC concentration was set as the half of the limit of quantification for statistical analyses.

Liquid chromatography–electrospray–high-resolution mass spectrometry was utilized for the measurement of 4*β*HC in rat serum at the Admescope Ltd. (Oulu, Finland)(Hautajarvi et al., 2018). The concentration of 4*α*-hydroxycholesterol was measured simultaneously to control for the sample storage conditions.

### Mononuclear Cell Isolation

Blood samples from the study subjects were collected into BD Vacutainer^®^ CPT Cell Preparation tubes with sodium citrate (BD, Franklin Lakes, NJ). After 30 min incubation at room temperature (RT), CPT tubes were centrifuged at 1800 × g for 30 min at 22°C, and the isolated mononuclear cells were collected. They were washed (1× PBS, 2 mM EDTA), centrifuged at 800 × g for 8 min at 22°C and resuspended in red blood cell lysis buffer (155 mM NH_4_Cl, 10 mM KHCO_3_, 2 mM EDTA). The cells were centrifuged at 200 × g for 5 min at 22°C, and the washes with lysis buffer were repeated if necessary. The purified mononuclear cells were washed twice with washing buffer by centrifuging at 200 × g for 5 min at 22°C. The cell suspension was lysed by suspending in RLT-buffer (RNeasy^®^ Mini Kit; Qiagen, Hilden, Germany) containing 1%-*β*-mercaptoethanol (Sigma-Aldrich). The cell lysate was stored at −70°C.

### Human *In Vivo* Mononuclear Cell RNA Extraction and qRT-PCR

For the homogenization, human mononuclear cell lysates were centrifuged at 16,000 × g for 2 min through QIAshredder (Qiagen) spin columns. From the homogenized flow through, total cellular RNA was isolated and purified using RNeasy^®^ Mini Kit columns and solutions (Qiagen) with DNase treatment according to the manufacturer’s instructions. The samples were stored at −70°C until use.

A 1 μg aliquot of the purified total cellular RNA from the cell samples was first converted to cDNA using Moloney-Murine Leukemia Virus (M-MuLV) reverse transcriptase and random hexamer primers of RevertAid First Strand cDNA synthesis kit (Thermo Fisher Scientific, Waltham, MA). For the qRT-PCR, the target genes were amplified in duplicates with iQ5 Multicolor Real-Time PCR Detection System iCycler using iQTM SYBR^®^ Green Supermix (Bio-Rad). The primers used are described in **Supplementary Table 1**. The results were analyzed by ΔΔCT algorithm with iQ5 Optical System Software (Bio-Rad). The relative gene expressions were calculated by normalization against human glyceraldehyde-3-phosphate dehydrogenase.

### Gene Expression Profiling of *In Vivo* Human Mononuclear Cells

The quality and integrity of the isolated human *in vivo* mononuclear cell RNA from the study volunteers (*n* = 12) was monitored by QIAxcel RNA QC Kit v2.0 (Qiagen) according to the manufacturer’s instructions. All the RNA Integrity Score values were above 7.4, indicating high-quality RNA with little degradation. RNA samples where first labeled using the Illumina Total PrepT RNA Amplification Kit (Life Technologies, Carlsbad, CA) according to the manufacturer’s instructions, with 350 ng of RNA per sample. The quality of all cRNA samples was controlled on 2100 Bioanalyzer RNA nano chips (Agilent Technologies) and quantified using a Nanodrop 2000 spectrometer (Thermo Fisher). A total of 750 ng of each sample was used in the Direct Hybridization Assay Workflow (Illumina, San Diego, CA) using HumanHT12_V4 expression beadchips (Illumina). The beadarrays were scanned using a HiScan instrument (Illumina). The work was carried out at the Core Facility of the Estonian Genome Center, University of Tartu, Estonia. Probe intensity and detection data were obtained using Illumina BeadStudio software, and further processed with GeneSpring GX 12.6 software (Agilent Technologies). The raw expression data was quality assessed, log2-transformed, and subjected to background adjustment and normalization. Genes were defined as differentially expressed if the fold change was at least 1.2-fold as compared to respective controls and statistically significant (*P* < 0.05, paired t-test and Benjamini and Hochberg false discovery rate). The complete data sets are available from the NCBI’s Gene Expression Omnibus (GEO) database (accession number GSE108100), and gene expression profiling data comply with the MIAME standard.

### Generation of Macrophages and Foam Cells for *In Vitro* qRT-PCR Experiments

Blood from one healthy female volunteer was collected into CPT tubes, and mononuclear cells were isolated as described above. The mononuclear cell suspension was centrifuged at 200 × g for 5 min at 22°C followed by suspension of the isolated cells in growth medium (1%-penicillin–streptomycin in DMEM, Sigma-Aldrich). The cells were plated and after 2-h adheration washed with PBS. Serum-free Macrophage-SFM medium (Invitrogen, Carlsbad, CA), 5 ng/ml Granulocyte-Macrophage-Colony-Stimulating-Factor (GM-CSF) and 1% penicillin–streptomycin was added to initiate the differentiation of monocytes into macrophages at 37°C, 5% CO_2_. The macrophage medium was changed every 2–3 days, and after 7 days the differentiation was confirmed by the phenotype analysis and by immunofluorescence staining with a mature-macrophage specific antibody MCA1122 (Bio-Rad, Raleigh, NC). For the qRT-PCR experiments, the macrophages were incubated for 18 h in growth medium without GM-CSF with 4*β*HC (1 µM, 10 µM, and 20 µM), 22R-hydroxycholesterol (22RHC; 10 µM and 20 µM, a positive control), rifampicin (10 µM and 20 µM), and ethanol (vehicle control for hydroxycholesterols) and dimethyl sulfoxide (vehicle control for rifampicin). The qRT-PCR was performed as described above.

Foam cells were generated by incubating macrophages (unexposed to study compounds) in LDL pool solution. The LDL pool was prepared by ultracentrifugation from human donor blood and acetylated with acetic anhydride (AcLDL pool). The differentiated macrophages cultured on 24-well-plates were washed 3 times with 1× PBS. AcLDL solution was added to a final concentration of 50 μg/ml diluted in growth medium without GM-CSF. In the rest of the wells plain growth medium without GM-CSF was added for reference control. Macrophages were incubated for 48 h at 37°C, 5% CO_2_ to produce foam cells. The phenotypic change was observed by light microscope and Oil Red O staining. The foam cells were incubated with the study compounds for 18 h, and qRT-PCR was performed as described above. The experiments on macrophages and foam cells were performed in four separate wells per experimental condition (*n* = 4).

### Cholesterol Efflux and Influx Experiments

Blood from one healthy female volunteer was collected, and macrophages were generated as described above. Foam cells for functional cholesterol transport experiments were generated from macrophages by incubating with [1*α*,2*α*(n)-^3^H]-cholesterol oleate-labeled acetylated LDL ([^3^H]-AcLDL). For the efflux and influx experiments, [^3^H]-AcLDL was added in each well to a final concentration of 50 μg/ml diluted in growth medium without GM-CSF and simultaneously exposed to a study compound or vehicle control. Macrophages were incubated for 24 h at 37°C, 5% CO2 to produce foam cells and then cells were washed 3 times with 1× PBS.

Then ApoAI and HDL_2_ acceptor pools prepared in efflux medium (DMEM, Sigma-Aldrich) were added into wells to a final concentration of 10 μg/ml and 50 μg/ml, respectively, and efflux medium without acceptors was used as control. Cells were incubated for 18 h at 37°C and 5% CO_2_. The mediums were collected and filtered through Nunc 96 Filter Plates (Fritted 96 DeepWell Plate, pore size 20 μm, Thermo Fisher) by centrifuging shortly at 100 × g. The cells were washed twice with 1× PBS and lysed in 0,1M NaOH solution by incubating for 2 h at RT on a plate stirrer. Liquid scintillation fluid Optiphase Supermix solution (Wallac, Perkin Elmer, Waltham, MA) was added to the mediums and cell lysates, and emissions were measured by scintillation and luminescence counter (1450 LSC & Luminescence Counter, Wallac, MicroBeta Trilux, Perkin Elmer). The protein concentrations of the cell lysates were determined according to the manufacturer’s instructions (DC Protein Assay Kit II, Bio-Rad). The influx and efflux experiments were performed in four separate wells per experimental condition (*n* = 4).

The cholesterol efflux (%) from foam cells was calculated using the following formula: [(counts per minute in medium)/(counts per minute in medium + counts per minute in cell lysate) x 100]. Furthermore, the efflux (%) was normalized to the cell protein concentration (%/μg). The cholesterol influx was calculated as the sum of counts per minute in medium and counts per minute in cell lysate normalized by cell protein content in each well (cpm/μg).

### Protocol of the Animal Experiment, RNA Extraction, qRT-PCR and Western Blots

Male Sprague Dawley rats were dosed with 40-mg/kg pregnenolone 16*α*-carbonitrile (PCN) or vehicle control (corn oil plus 30% dimethyl sulfoxide) for 1, 3, or 6 days (five rats per group) as previously described (Rysa et al., 2013). The rats dosed with PCN *vs.* vehicle control are an established model for the study of the effects of PXR activation. The dosing was randomized; no blinding was undertaken. At the end of the experiment, the rats were decapitated, and the organs were immersed in liquid nitrogen and stored at −70°C. The experimental designs were approved by the national Animal Experiment Board in Finland. Animal studies are reported in compliance with the ARRIVE guidelines (Kilkenny et al., 2010; Mcgrath and Lilley, 2015). The investigation conforms to the Guide for the Care and Use of Laboratory Animals as adopted and promulgated by the US National Institutes of Health.

Total RNA was isolated using RNAzol reagent (Sigma-Aldrich) according to the manufacturer’s protocol. One microgram of RNA was reverse transcribed to produce complementary DNA using p(dN)6 random primers and RevertAid reverse transcriptase. The qRT-PCR reactions were performed using FastStart Universal SYBRGreen Master Mix or FastStart Universal Probe Master Mix (Roche, Basel, Switzerland). The sequences of the rat primers are presented in **Supplementary Table 2**. Fluorescence values of the qRT-PCR products were corrected with the fluorescence signals of the passive reference dye (ROX; Roche). The RNA levels of target genes were normalized against the 18S control levels using the ΔΔCT method.

Total protein was extracted from the left ventricle of the heart of vehicle or PCN-treated rats (5 rats per group) in 0.1 M phosphate buffer supplemented with Halt™ protease and phosphatase inhibitor cocktail. Protein concentrations were measured using the Bradford protein assay. Aliquot of 25 µg protein was separated on a 10% SDS-polyacrylamide gel electrophoresis and transferred to nitrocellulose membrane. Blots were blocked for 1 h at room temperature with 1% Milk (ECL prime™ blocking reagents; GE Healthcare, Chicago, IL, United States) in TBS buffer containing 0.05% Tween 20, then incubated overnight at +4°C with ABCA1 rabbit polyclonal antibody (NB400-105SS, 1:500; Novus Biologicals, Abingdon, UK), ABCG1 rabbit polyclonal antibody (NB400-132SS,1:1,000; Novus) and mouse monoclonal anti-*β*-actin antibody (A1978, 1:5,000; Sigma-Aldrich), antibodies were diluted in TBS buffer containing 5% milk and 0.05% Tween 20. Then membranes were washed 5 times 5 min with TBS buffer containing 0.1% Tween 20 before finally incubated with secondary antirabbit antibody (SC-2004, 1:1,500; Santa Cruz Biotechnology) or antimouse antibody (NA931, 1:10,000; Amersham ECL, GE Healthcare) 1 h at room temperature. The immunoreactive bands were detected by chemiluminescence (ECL start Western Blotting Detection Reagents; GE Healthcare) and quantified (Odyssey^®^ FC imaging system; LI-COR Biosciences, Lincoln, NE, USA). The amount of the ABCA1 and ABCG1 protein was normalized against the *β*-actin.

### Statistical Analyses

The parameters of the volunteer study were compared across treatments by paired two-tailed Student’s t-test. The intraindividual ratio (rifampicin to placebo) of the expression of gene transcripts in mononuclear cells *in vivo* was analyzed with one-sample t-test. Unpaired two-tailed Student’s t-test (when two groups) and ANOVA with Dunnett’s *post hoc* test (when more than two groups) were used to analyze the cell and animal experiments. The statistics were calculated using an Excel template provided in Microsoft Office (Microsoft, Redmond, WA, USA), SPSS (IBM, Armonk, NY, USA) and GraphPad Prism (GraphPad Software, San Diego, CA, USA). No outliers were excluded in any of the experiments or analyses. *P* < 0.05 was considered statistically significant.

## Results

### Effect of Rifampicin Treatment on Serum Oxysterols and Cholesterol in Healthy Volunteers

Twelve healthy subjects (three women, nine men) participated in the clinical study (Rysa et al., 2013). The mean age was 24 years (SD ± 5.2; range 19–38 years), the mean weight was 73 kg (SD ± 10.8; range 57–98 kg), and the mean BMI was 24.0 (SD ± 2.8; range 20.6–28.9 kg/m^2^).

Rifampicin 600 mg once per day for a week increased serum 4*β*HC concentration when compared with placebo dosing as expected (**Table 1**). The mean intraindividual induction was 2.2-fold (range 1.14–3.72). Also 4*β*HC concentration normalized by total cholesterol concentration was elevated to a similar extent. Serum 25HC and 27HC concentrations were not affected by rifampicin. Cholesterol, HDL, LDL, and triglycerides were not changed statistically significantly by rifampicin.

**Table 1:** Effect of rifampicin treatment on serum 4*β*-hydroxycholesterol, 25-hydroxycholesterol, 27-hydroxycholesterol and cholesterol values (*n* = 12). Data are presented as mean ± SD. The oxysterol to cholesterol ratios are presented as molar ratios × 10 000. ***P < 0.0001; paired two-tailed Student’s t-test.

	Rifampicin	Placebo
4*β*HC (ng/ml)	72.0 ± 21	35.0 ± 12^***^
4*β*HC/Cholesterol	0.42 ± 0.09	0.22 ± 0.07^***^
25HC (ng/ml)	2.83 ± 1.1	2.89 ± 1.2
25HC/Cholesterol	0.016 ± 0.005	0.018 ± 0.006
27HC (ng/ml)	42.3 ± 8.8	42.3 ± 8.3
27HC/Cholesterol	0.26 ± 0.08	0.27 ± 0.10
Cholesterol (mmol/l)	4.22 ± 0.8	4.03 ± 0.7
LDL (mmol/l)	2.38 ± 0.7	2.21 ± 0.5
HDL (mmol/l)	1.44 ± 0.2	1.36 ± 0.2
Triglycerides (mmol/l)	0.69 ± 0.3	0.77 ± 0.3

### The Effect of Rifampicin Treatment on Mononuclear Cell Gene Expression in Healthy Volunteers

**Figure 1:**
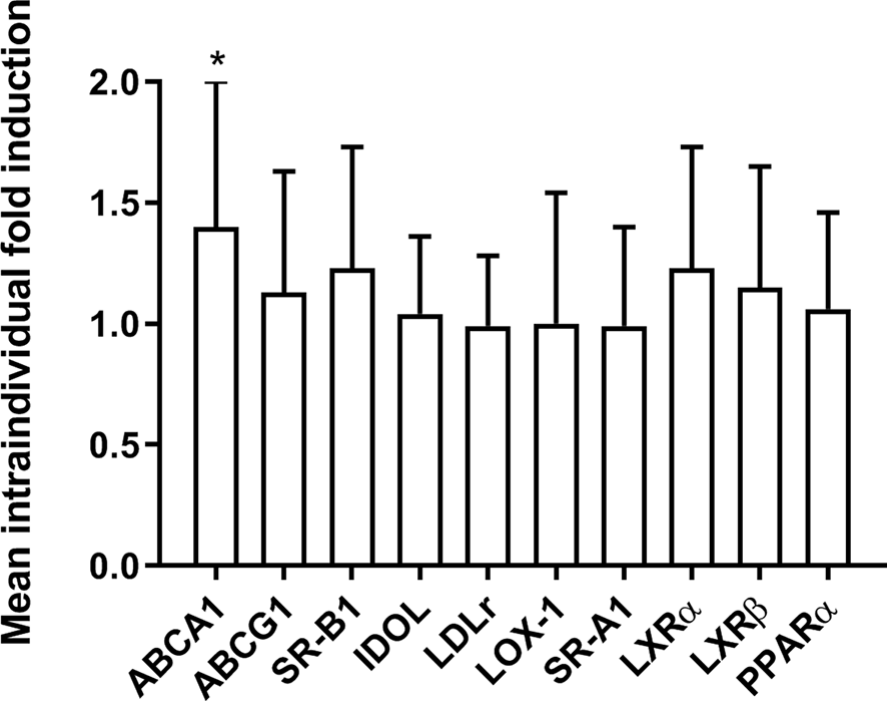
The effect of rifampicin (600 mg per day for one week) *vs.* placebo on the expression of lipid transporters and receptors involved in their regulation in the mononuclear cells of healthy volunteers *in vivo* (*n* = 12) as measured with quantitative RT-PCR. The data are presented as the mean intraindividual ratio (rifampicin to placebo) of transcript expression levels +SD. *P < 0.05; one-sample t-test.

The qRT-PCR analyses for mRNA expression of cholesterol transporters and their regulators were performed in mononuclear cells isolated from the 12 volunteers. ABCA1 expression was induced by rifampicin dosing when analyzed with one-sample *t*-test (intraindividual fold induction 1.40, *P* = 0.042). The expression of other genes did not change significantly (**Figure 1**). The quantitative measurement of PXR and ABCG5 expression was unsuccessful due to low expression level, but the expression was detectable with conventional RT-PCR in all subjects. ABCG8 was not detected.

In the whole genome gene expression analysis of the 12 study subjects’ mononuclear cells with the Illumina Human HT-12 expression beads, only one gene was differentially expressed when conventional false discovery rate correction was used; ornithine decarboxylase antizyme 2 (OAZ2) was downregulated by rifampicin treatment when compared with the placebo arm. When less stringent statistical analysis was employed, the expression of 15 genes was significantly dysregulated (*P* < 0.05 without false discovery rate correction) by rifampicin treatment (**Supplementary Table 3**).

### The Incubation of Foam Cells With 4*β*HC Induces Cholesterol Efflux and Represses Cholesterol Influx

**Figure 2:**
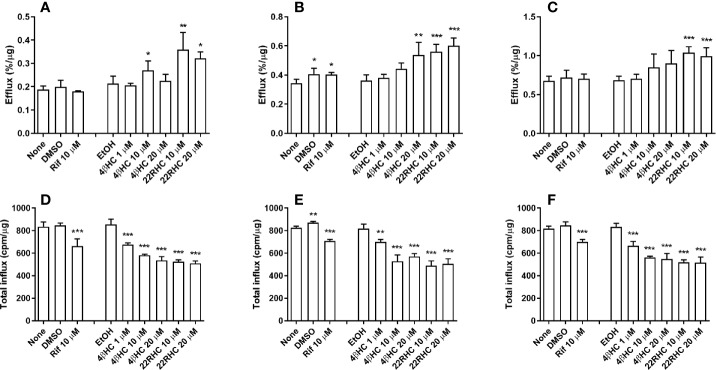
Effect of rifampicin and oxysterols on the cholesterol efflux (upper panels **A**–**C**) and the total cholesterol influx (lower panels **D**–**F**) in human primary monocyte–macrophage generated foam cells *in vitro* (normalized by protein content). Experiments without acceptors (panels **A, D**) and with acceptors (ApoAI, panels **B, E**; HDL_2_, panels **C, F**) are shown. The experiments were performed in four separate wells per experimental condition (n = 4). The results are presented as mean + SD. 22RHC is a positive control, and DMSO and EtOH are vehicle controls for rifampicin and oxysterols, respectively. *P < 0.05, **P < 0.01, and ***P < 0.001 *versus* vehicle control; ANOVA with Dunnett’s *post hoc* test.

The functional cholesterol transport was studied in foam cells generated from human primary monocyte–macrophages *in vitro*. Experiments were performed both with and without acceptors (ApoAI and HDL_2_). Macrophages were simultaneously exposed to a study compound and 50 μg/ml ^3^H-labeled acetylated LDL for 24 h. Cholesterol efflux was induced by 4*β*HC, especially when ApoAI was used as acceptor. The efflux facilitated by ApoAI was induced 1.5-fold at 20 µM 4*β*HC concentration (*P* = 0.0014,; **Figure 2B**). HDL_2_-facilitated efflux was elevated 1.3-fold at 20 µM concentration (*P* = 0.078 statistically not significant; **Figure 2C**). Efflux to medium without acceptors was not affected. Rifampicin did not have an effect on cholesterol efflux. 22R-hydroxycholesterol used as a positive control induced cholesterol efflux as expected.

Cholesterol influx was affected by oxysterols even more pronouncedly than efflux. The 1 µM concentration of 4*β*HC repressed influx by 21% (*P* < 0.001), and 20 µM concentration caused repression of 37% (*P* < 0.0001) (**Figure 2D**). The maximal repression caused by 22RHC was 40% (*P* < 0.0001). While efflux to medium was significantly increased by ApoAI and HDL_2_ acceptors as expected, the suppressing effect of oxysterols on the cholesterol influx was not affected by ApoAI and HDL_2_ (**Figures 2E, F**). Rifampicin repressed cholesterol influx when normalized to protein content; this is explained by an increase in protein content elicited by rifampicin. The suppressing effect of oxysterols on influx was not affected by protein content normalization.

### The Effect of Acetylated LDL Incubation on the Expression of Cholesterol Transporters

In *in vitro* experiments with human primary monocyte–macrophages (without AcLDL incubation) and foam cells (with prior AcLDL incubation), foam cells had noticeably higher expression of efflux transporters ABCA1, ABCG1, and SR-B1 when compared with macrophages (**Supplementary Figure 1A**). AcLDL treatment had transporter-specific effects on the influx transporters (**Supplementary Figure 1B**). Both LDL receptor and lectin-like oxidized LDL receptor-1 (LOX-1) were strongly repressed. The inducible degrader of the LDL receptor (IDOL), a major regulator of LDL receptor in macrophages and an LXR target (Hong et al., 2014), was induced in foam cells compared with macrophages. The expression of cluster of differentiation 36 (CD36) was induced by AcLDL treatment.

### The Expression of Efflux Transporters ABCA1 and ABCG1 Is Induced by 4*β*HC

**Figure 3:**
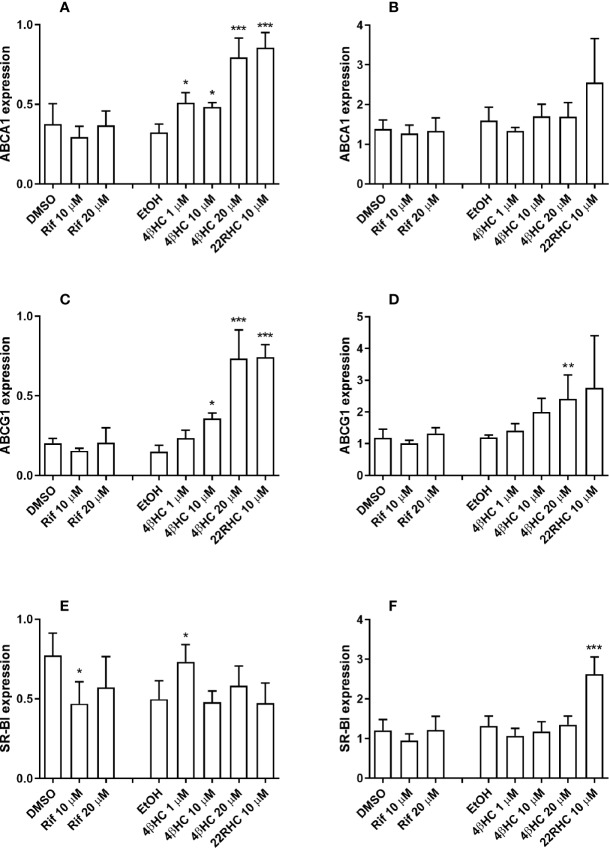
Effect of rifampicin and oxysterols on the mRNA expression of cholesterol efflux transporters in human primary monocyte–macrophages and foam cells *in vitro* measured with quantitative RT-PCR. Panels on the left **(A, C, E)** show the results of the experiments in macrophages and the panels on the right **(B, D, F)** display the results of the experiments in foam cells. The experiments were performed in four separate wells per experimental condition (*n* = 4). Data are presented as the mean + SD. *P < 0.05, **P < 0.01, and ***P < 0.001; ANOVA with Dunnett’s *post hoc* test.

The incubation with 4*β*HC caused dose-dependent induction of efflux transporters ABCA1 and ABCG1 mRNAs in macrophages that reached statistical significance at 1 µM and 10 µM concentrations, respectively (**Figures 3A, C**). The maximum induction with 20 µM concentration of 4*β*HC was 2.5-fold for ABCA1 (*P* < 0.0001) and 4.9-fold for ABCG1 (*P* < 0.0001). In foam cells, ABCG1 was induced by 4*β*HC (maximum induction 2.0-fold, *P* = 0.0058), while ABCA1 was not induced (**Figures 3B, D**). It is noteworthy that 4*β*HC was able to further increase the expression of ABCG1 even in the setting of already AcLDL-induced ABCG1 expression.

### The Expression of Influx Transporter LOX-1 Is Repressed and IDOL Is Induced by 4*β*HC

**Figure 4:**
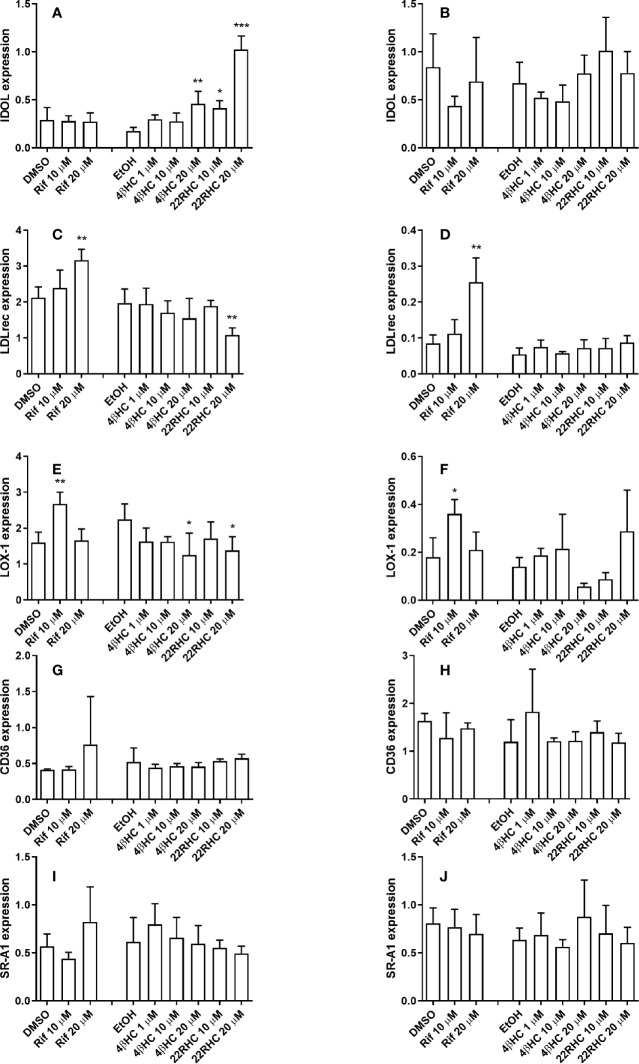
Effect of rifampicin and oxysterols on the mRNA expression of cholesterol influx transporters in human primary monocyte–macrophages and foam cells *in vitro* measured with quantitative RT-PCR. Panels on the left **(A, C, E, G, I)** show the results of the experiments in macrophages and the panels on the right **(B, D, F, H, J)** display the results of the experiments in foam cells. The experiments were performed in four separate wells per experimental condition (*n* = 4). Data are presented as the mean + SD. *P < 0.05, **P < 0.01, and ***P < 0.001; ANOVA with Dunnett’s *post hoc* test.

The incubation with 4*β*HC caused dose-dependent repression of LOX-1 in macrophages (reaching statistical significance at 20 µM concentration (*P* = 0.016); **Figure 4E**). The maximum LOX-1 repression was 0.56-fold (*P* = 0.037) in macrophages. IDOL expression was dose-dependently induced in macrophages by 4*β*HC reaching statistical significance at 20 µM concentration (2.6-fold, *P* = 0.0012; **Figure 4A**). There were no effect of 4bHC on the expression of other transcripts measured (**Figures 4B–D, F–J**).

### The Effect of 22RHC and Rifampicin on the Expression of Cholesterol Transporters

The incubation of macrophages with 22RHC, a positive control, induced ABCA1 and ABCG1 as expected, and also induced IDOL and repressed LDLr and LOX-1 (**Figures 3** **and** **4**). In foam cells, 22RHC induced SR-B1 (**Figure 3F**). We also measured the effect of rifampicin on the expression of cholesterol transporters. Rifampicin at 10 µM (but not at 20 µM) concentration caused a small decrease in SR-B1 mRNA expression in macrophages (**Figure 3E**). Rifampicin induced LDLr at 20 µM in macrophages and foam cells (**Figure 4**). LOX-1 expression was induced by rifampicin at 10 µM (but not 20 µM) concentration in macrophages and foam cells.

### Treatment of Rats With a PXR Agonist Elevates 4*β*HC and Induces LXR Targets in Peripheral Tissues

To further investigate the *in vivo* effects of PXR activation on LXR targets in peripheral tissues, rats were dosed with PCN, a rat PXR agonist, *versus* vehicle control for 1, 3, or 6 days. The serum concentration of 4*β*HC was elevated already on Day 1 (1.3-fold; 66.0 ± 13.9 *vs.* 85.1 ± 21.1 ng/ml, *P* < 0.05) and further increased on Day 6 (8.7-fold; 55.2 ± 5.8 *vs.* 480 ± 95 ng/ml, *P* < 0.05) by PCN dosing. PCN did not affect the concentration of 4*α*-hydroxycholesterol. The mean serum 4*α*-hydroxycholesterol concentration was 13.4 ± 3.0 ng/ml.

**Figure 5:**
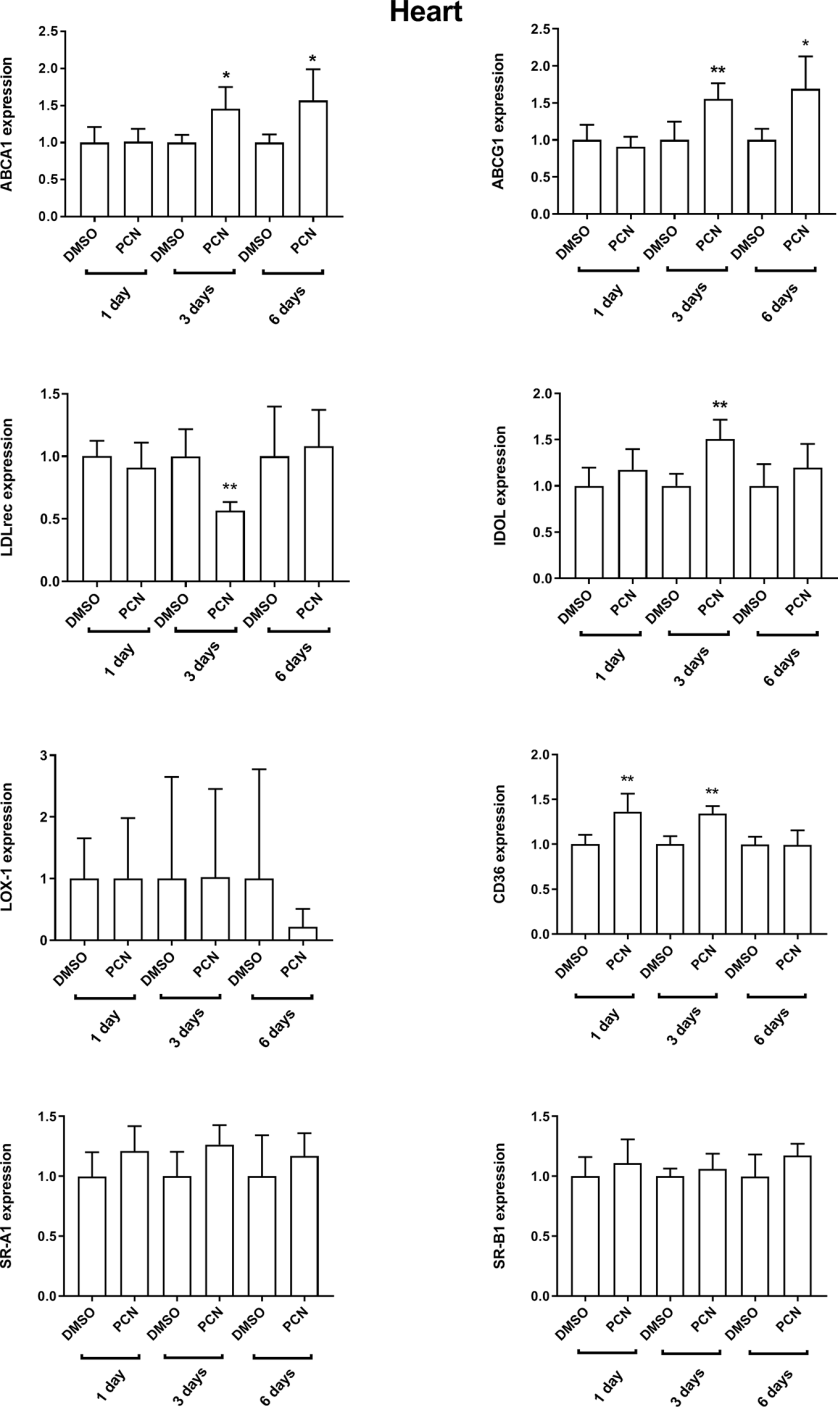
Effect of intraperitoneal pregnenolone 16α-carbonitrile (a rat PXR agonist) 40 mg/kg *versus* vehicle control (DMSO) for 1, 3, or 6 days on the relative mRNA expression of cholesterol transporters in rat heart *in vivo* as measured with quantitative RT-PCR. The experiments were performed with five rats per experimental condition (*n* = 5). Data are presented as the mean + SD. *P < 0.05, and **P < 0.01 unpaired two-tailed Student’s t-test.

The mRNA expression of efflux and influx transporters was measured in rat peripheral tissues (**Figure 5** and **Supplementary Figures 2–5**). ABCA1 and ABCG1 were induced by PCN in the heart (days 3 and 6, **Figure 5**). ABCA1 was induced in the muscle (days 1 and 6, **Supplementary Figure 2**). LDLr was repressed and IDOL, the negative regulator of LDLr, was induced in the heart on day 3. To interrogate the regulation of LDLr, we calculated the ratio of LDLr to IDOL (LDLr/IDOL) which was repressed by PCN dosing on day 3 in the heart (**Supplementary Figure 5**). In the muscle tissue, LDLr tended to be suppressed and IDOL tended to be induced while LDLr/IDOL was suppressed on days 3 and 6. Although LDLr and IDOL were not affected by PCN in white adipose tissue (**Supplementary Figure 3**), LDLr/IDOL ratio was repressed on day 6. In adrenal gland, LDLr was repressed on day 1 and IDOL on day 6 (**Supplementary Figure 4**). LDLr/IDOL ratio was downregulated on day 1 and upregulated on day 3 in adrenal gland. The expression of CD36 was induced in the heart (day 1 and day 3) and muscle (day 1), and repressed in the adrenal gland (day 6).

**Figure 6:**
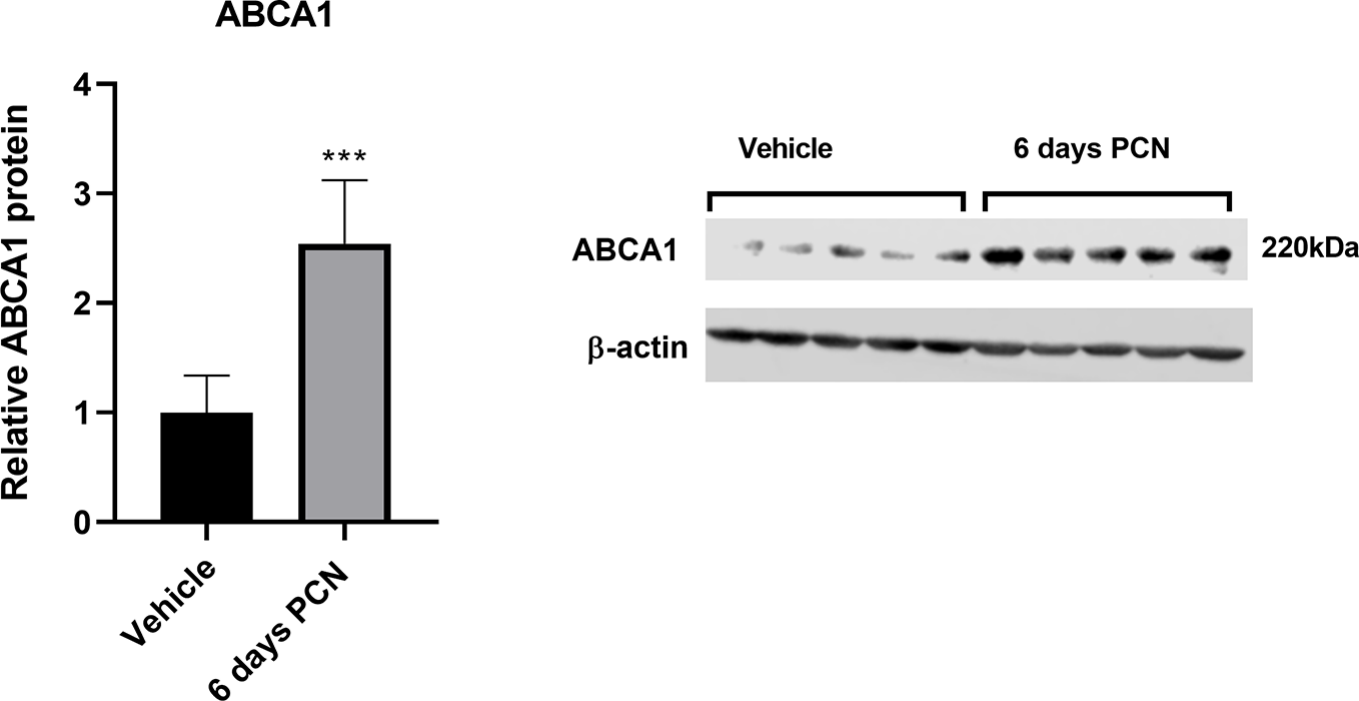
Effect of intraperitoneal pregnenolone 16*α*-carbonitrile (a rat PXR agonist) 40 mg/kg *versus* vehicle control (DMSO) for 6 days on the ABCA1 protein expression in the rat heart (left ventricle). The experiments were performed with five rats per experimental condition (*n* = 5). Data are presented as the mean + SD. ***P < 0.001 *versus* vehicle control; unpaired two-tailed Student’s t-test.

The protein expression of ABCA1 and ABCG1 in the left ventricle of the rat heart was measured on day 6. ABCA1 protein was induced 2.5-fold in the heart (*P* = 0.0009; **Figure 6**), while the trend of the 1.4-fold ABCG1 induction was statistically not significant (*P* = 0.051; **Supplementary Figure 6**).

## Discussion

This study demonstrates that physiologically relevant concentrations of 4*β*HC, an LXR*α* and *β* agonist, have a repressing effect on cholesterol influx and inducing effect on cholesterol efflux of human macrophages and foam cells *in vitro*. The efflux facilitated by ApoAI was augmented more than the efflux to HDL_2_. The mRNA expression of efflux transporters ABCA1 and ABCG1 was induced by 4*β*HC; this is a novel finding. As these transporters are the main efflux transporters in macrophages and foam cells, their induction explains the enhanced transport of cholesterol. Furthermore, the expression of ABCA1 mRNA in mononuclear cells of healthy volunteers *in vivo* was induced by rifampicin dosing. As rifampicin, efficient PXR agonist, did not directly induce cholesterol efflux transporters *in vitro*, the rifampicin induction of ABCA1 is most likely mediated *via* increased circulating 4βHC that activates LXR in mononuclear cells. The concentration of circulating 4*β*HC was induced more than twofold by rifampicin administration as expected.

In addition to the enhanced efflux, 4*β*HC and 22RHC had a profound suppressing effect on cholesterol influx. The incubation with 4*β*HC and 22RHC caused dose-dependent repression of influx transporter LOX-1 in macrophages. To our knowledge, this is the first time that any oxysterol has been shown to modulate the expression of LOX-1. The expression of IDOL, a major regulator of macrophage LDL receptor, was dose-dependently induced in macrophages by 4*β*HC. Somewhat surprisingly, the expression of LDLr was not affected by 4βHC regardless of the induction of IDOL. Incubation of macrophages with 22RHC induced IDOL and repressed LDLr and LOX-1. SR-AI and CD36 are generally considered to be responsible for the majority of the uptake of acetylated and oxidized LDL in macrophages (Kunjathoor et al., 2002; Moore et al., 2013). We demonstrate here that the expression of CD36 and SR-A1 is not affected by oxysterols 4*β*HC and 22RHC. Our results indicate that LOX-1 may have a more significant role in acetylated LDL uptake than previously recognized.

It is well-known that oxysterols induce cholesterol efflux by activating LXR in macrophages (Schwartz et al., 2000; Venkateswaran et al., 2000; Sparrow et al., 2002). Much less is known about the suppression of LDL influx by oxysterols. The research performed by Kruth et al. have shown that the intake of ^125^I-labeled native LDL (but not the intake of ^125^I-labeled acetylated LDL) is suppressed by 22RHC and a synthetic LXR agonist T0901317 in human macrophages (Buono et al., 2007; Kruth, 2011). The intake of native LDL was considered to occur through receptor-independent, fluid-phase pinocytosis. The intake of ^125^I-acetylated LDL was saturable which is consistent with receptor-mediated endocytosis. In HepG2 cells, incubation with synthetic (GW3965, T0901317, and SB742881) and an endogenous (24(S),25-epoxycholesterol) LXR agonists decreased cholesterol influx (Aravindhan et al., 2006). Furthermore, LXR activation by synthetic LXR ligands suppressed the uptake of boron-dipyrromethene-labelled LDL and DiI-LDL in HepG2 cells and mice macrophages (Zelcer et al., 2009; Hong et al., 2014). Therefore, there are previous data indicating that LXR activation represses cholesterol influx but no prior study had explored the effect of 4*β*HC.

In the whole genome gene expression analysis of the volunteers’ mononuclear cells, only one gene was affected (OAZ2 downregulated) by rifampicin dosing when false discovery rate correction was used. OAZ2 encodes ornithine decarboxylase antizyme 2 that negatively regulates ornithine decarboxylase activity involved in polyamine biosynthesis and uptake (Perez-Leal and Merali, 2012). Polyamines block the potassium outflow of the inward-rectifier potassium channels such as KCNJ2 (Baronas and Kurata, 2014) which was found to be the second-mostly suppressed gene in our analysis. It can be hypothesized that OAZ2 suppression leads to an increase in intracellular polyamine levels which enhance the KCNJ2-mediated uptake of potassium ions, and perhaps KCNJ2 expression is then suppressed in a counter-regulatory loop. In macrophages, KCNJ2 is known to have a role in lipid uptake and foam cell formation by modulating the expression of SR-A, SR-B1, and CD36 (Zhang et al., 2016). It is not known if LXR is involved in the regulation of OAZ2 or KCNJ2.

The PXR activation by rifampicin increases the concentration of circulating 4*β*HC, and we demonstrate that 4*β*HC induces the expression of known LXR targets (ABCA1, ABCG1, and IDOL) in macrophages *in vitro*. To provide evidence that PXR activation induces these LXR targets also *in vivo*, we dosed rats with PCN, a prototypical rodent PXR agonist. PXR activation is not expected to directly affect the expression of LXR targets ABCA1, ABCG1, and IDOL. Furthermore, PXR is expressed in rat liver, intestine, and kidney, but not in the heart (Zhang et al., 1999). Dosing with PXR ligand PCN increased the circulating 4*β*HC in rats similarly to human volunteers with rifampicin dosing. In accordance with the elevated serum 4*β*HC, we detected mRNA induction of ABCA1 and ABCG1 in the heart and the induction of ABCA1 in the muscle. ABCA1 was also clearly induced at the protein level in the rat heart. These findings are analogous to the induction of ABCA1 expression we detected in human mononuclear cells *in vivo* by PXR agonist rifampicin.

In addition, LXR target IDOL tended to be induced in the rat heart and muscle. In contrast, the repression of IDOL was detected in the adrenal gland. This probably reflects the unique role of adrenal gland as a site of steroidogenesis requiring cholesterol transport to the adrenal cortex from the circulation. As IDOL is a major regulator of LDLr expression, we calculated the ratio of LDLr to IDOL to further evaluate the regulation of LDLr. The LDLr/IDOL ratio was clearly repressed by PCN dosing in muscle. Altogether, our *in vivo* rat experiment provides evidence that treatment with PXR ligand induces the expression of LXR targets in peripheral tissues most probably through hepatic production and secretion of LXR activator 4*β*HC (*i.e.* hepatic PXR–circulating 4βHC–LXR pathway).

Our study has some limitations; although we did show the effect of 4*β*HC on the influx and efflux transporters in foam cells generated from the human primary monocyte–macrophages in the mRNA level and the functional level, we did not measure the protein expression. However, as the effect of 4*β*HC on the mRNA expression and the protein function (the influx and efflux of cholesterol) were congruent *in vitro*, and in the rat heart we present the induction of both mRNA and protein of ABCA1 and ABCG1 *in vivo*, we consider that the lack of protein expression data *in vitro* does not hamper the interpretation of the results. As our aim was to explore the effect of 4*β*HC on cholesterol transporters in monocyte–macrophages and foam cells, we did not study the effect of 4*β*HC on the formation of foam cells which would be an interesting topic in itself. Also, the exact mechanism of 4*β*HC-mediated repression of LOX-1 requires further study.

The average serum concentration of 4*β*HC was 87 nM during placebo and 179 nM during rifampicin arm but up to 1,500 nM concentrations have been reported in patients using enzyme-inducing antiepileptics (Bodin et al., 2001). In simulation studies even a low concentration of a ligand in the presence of multiple other low-affinity ligands can have profound effects on the target gene expression in a multilinked regulatory network (Kolodkin et al., 2013). A mixture of LXR ligands is present in the body, and more than doubling the concentration of an agonist could plausibly be a signal with significant downstream effects.

The hepatic PXR–circulating 4*β*HC–peripheral LXR pathway could have important implications for lipid homeostasis. Circulating 4*β*HC might serve as a signaling molecule informing the extrahepatic tissues about the cholesterol concentration in liver. This notion is supported by the finding in swine that a high-cholesterol diet resulting in doubling of the hepatic cholesterol concentration increased 4*β*HC 18-fold in serum and 21-fold in cardiac left ventricle (Shimabukuro et al., 2016). LXR agonists are known to activate reverse cholesterol transport (RCT), a beneficial pathway transporting cholesterol from periphery to the liver and intestine for excretion (Temel and Brown, 2015). The activation of LXR and enhanced RCT protects against lipid deposition in vascular endothelium (Lee and Tontonoz, 2015). Therefore, as LXR agonist, the increased 4*β*HC concentration could be hypothesized to result in the activation of RCT and attenuation of pro-atherogenic actions associated with PXR activation in liver and intestine (Zhou, 2016). Although *in vitro* experiments indicate that PXR may regulate 25HC and 27HC production, the circulating levels of these oxysterols with important immunologic actions were not increased by rifampicin *in vivo*.

Although PXR activation by short-term rifampicin dosing did not affect cholesterol levels to a significant degree in our study, the treatment with PXR agonists such as carbamazepine, phenobarbital, and phenytoin is associated with increased HDL while total cholesterol remains constant (Nikkila et al., 1978; O’neill et al., 1982). CYP content of liver biopsy samples correlates with HDL in patients with epilepsy (Luoma et al., 1980), and in psychiatric patients CYP3A activity correlates strongly with HDL (Choong et al., 2013). Phenytoin increased HDL and especially HDL_2_ but not HDL_3_, total cholesterol, or LDL in a placebo-controlled parallel trial in patients with low HDL (Miller et al., 1995). Occupational exposure to pesticide lindane, a potent PXR agonist (Kojima et al., 2011), associates with remarkably high HDL concentrations (Carlson and Kolmodin-Hedman, 1972). Thus, there are indirect evidence in humans for the significance of CYP3A activity in cholesterol, and especially HDL, metabolism suggesting that 4*β*HC may have a role in activating reverse cholesterol transport *in vivo*. As the half-life of HDL is a few days (Blum et al., 1977), one-week dosing of rifampicin, as in our study, is too short a time period for the full effect of 4*β*HC elevation on HDL level to be manifested. Of note, 4*β*HC levels are low in conditions associated with low HDL levels such as metabolic syndrome, obesity, and nonalcoholic fatty liver disease (Tremblay-Franco et al., 2015; Woolsey et al., 2015). Furthermore, women are known to have higher 4*β*HC and HDL compared with 4*β*HC and HDL levels in men (Diczfalusy et al., 2011; Wang et al., 2011).

In conclusion, PXR activation-elevated 4*β*HC is a signaling molecule that activates LXR to repress cholesterol influx and induce efflux in macrophages. The activation of hepatic PXR results in the upregulation of LXR targets in peripheral tissues. A multitude of environmental and occupational chemicals as well as pharmaceuticals and herbal remedies have PXR-activating properties (Hukkanen, 2012; Hukkanen et al., 2014). Thus, the hepatic PXR–circulating 4*β*HC–peripheral LXR pathway links the exposure to xenobiotics and the regulation of cholesterol transporters. The physiological role of this pathway could be to enhance the excretion of excess cholesterol and attenuate the progression of atherosclerosis. The studies investigating the functional consequences of PXR–4*β*HC–LXR pathway *in vivo* on reverse cholesterol transport in humans are warranted.

## Data Availability Statement

The expression dataset generated for this study can be found in the GEO database as a data set GSE108100 https://www.ncbi.nlm.nih.gov/geo/query/acc.cgi.

## Ethics Statement

This study was carried out in accordance with the Guideline for Good Clinical Practice as recommended by the ethics committee of the Northern Ostrobothnia Hospital District and the Finnish Medicines Agency with written informed consent from all subjects. All subjects gave written informed consent in accordance with the Declaration of Helsinki. The protocol was approved by the ethics committee of the Northern Ostrobothnia Hospital District and the Finnish Medicines Agency. This study was carried out in accordance with the recommendations of Guide for the Care and Use of Laboratory Animals as adopted and promulgated by the US National Institutes of Health. The protocol was approved by the national Animal Experiment Board in Finland.

## Author Contributions

TS, MS, PK, JRy, JHa, and JHu participated in the research design. TS, PK, JRy, HN, and JRo conducted experiments. TH, HH, AT, and MO contributed new analytic tools. TS, PK, JRy, HN, and JRo performed data analysis. JHu performed the clinical study. TS and JHu wrote the initial drafts of the manuscript. All authors reviewed and edited the manuscript.

## Funding

The study was financially supported by the grants from the Academy of Finland [Grants 286743, 276747]; the Novo Nordisk Foundation [Grants NNF14OC0010653, NNF15OC0015846]; the Duodecim Society of Oulu; the Finnish Medical Foundation; the Finnish Foundation for Cardiovascular Research; the Northern Finland Health Care Support Foundation; and the Diabetes Research Foundation.

## Conflict of Interest

HH and AT are employed by company Admescope Ltd., which has developed the commercially available 4*β*- and 4*α*-hydroxycholesterol analysis method.

The remaining authors declare that the research was conducted in the absence of any commercial or financial relationships that could be construed as a potential conflict of interest.

## Abbreviations

4*β*HC, 4*β*-hydroxycholesterol; 22RHC, 22R-hydroxycholesterol; 25HC, 25-hydroxycholesterol; 27HC, 27-hydroxycholesterol; ABC, ATP-binding cassette transporter; AcLDL, acetylated LDL; CD36, cluster of differentiation 36; CYP, cytochrome P450; EtOH, ethanol; HDL, high-density lipoprotein cholesterol; IDOL, inducible degrader of the LDL receptor; LDL, low-density lipoprotein cholesterol; LDLr, LDL receptor; LOX-1, lectin-like oxidized LDL receptor-1; LXR, liver X receptor; PXR, pregnane X receptor; RCT, reverse cholesterol transport; SR, scavenger receptor.
