# Supplementary material for: 4β-Hydroxycholesterol Signals From the Liver to Regulate Peripheral Cholesterol Transporters
Source: Front Pharmacol. 2020 Mar 27;11:361. doi: 10.3389/fphar.2020.00361 (PMC7118195; doi:10.3389/fphar.2020.00361)
Supplement: Supplementary file 1 [file DataSheet_1.pdf]

SUPPLEMENTARY TABLE 1 Human oligonucleotide sequences used for qRT-PCR and conventional RT-PCR analyses with annealing temperatures (Ta).

**Gene**	**Forward primer (5' - 3')**	**Reverse primer (5' - 3')**	**T_a (°C)**
abca1	GAACATTACAGCAGATCAAGAAATCC	ACTTTGGGAGAGAGAGGTTGTGA	60
abcg1	CAGGAAGATTAGACACTGTGG	GAAAGGGGAATGGAGAGAAGA	59
abcg5	GTGGTTTATAGTCCCTTGCTCTTAC	CACACGAGTCTCCCATAGGT	61
abcg8	GTGGGCAACATGTACGTG	CAGAGGTGGGTTCGTCG	61
srb1	TTGAACTTCTGGGCAAATG	TGGGGATGCCTTCAAACAC	60
lxrA	TCAGCATCTTCTCTGCAGACCGG	TCATTAGCATCCGTGGGAACA	60
lxrB	GCTAACAGCGGCTCAAGAACTAA	GCTCCGTGAAGTGGGCAAA	60
pxr	TGCGCATCCAGGACATAC	TCTCGGAGGTGTCACCC	59
gapdh	GAGTCAACGGATTTGGTCGT	GACAAGCTTCCCGTTCTCAG	60
cd36	GCAAGTCCTGATGTTTCAG	GGTCCCAGTCTCATTAAGC	62
ldlr	ACCTGTCCCAGAGAATGATCT	TCGGTCCAGTAGATGTTGC	60
sra1	GGATACCCAGGTGTTCAAG	CAGCCATATTGGACCAGTA	60
idol	CACCCAGTCAGGAAAGAAT	CTGTGTCACACCTGTAGAA	64
lox1	GCCTGATGAGAAGTCAAATG	GTTAGGTTTGCTTGCTCTTG	62

SUPPLEMENTARY TABLE 2 Rat oligonucleotide sequences used for qRT-PCR analyses with annealing temperatures (T_a).

Gene	Forward primer (5' - 3')	Reverse primer (5' - 3')	T_a (°C)
abca1	CAAGAGATGGACCTTGTGCG	TGGGGACTGAACATCCTCTG	60
abcg1	GATGAAGGCAGACGGGAGA	AACAGGAGGGTTGTTGACCA	60
lox-1	CTATCCTTTCTTGGGTGTAAAAC	TGCTTCTGGTCTTTGTCTCTG	60
idol	GGCCATACTGTGTGCTGTGA	ATGTTCCACACGTGATCTGC	60
ldlr	TGCTACTGGCCAAGGACAT	CTGGGTGGTCGGTACAGTG	60
srb1	GGTGCCCATCATTTACCAAC	GCGAGCCCTTTTTACTACCA	60
cd36	GCCTCCTTTCCACCTTTTGT	GATTCAAACACAGCATAGATGGA	60
sra1	Rn01488115_m1 (TaqMan, Thermo Fisher)		60

SUPPLEMENTARY TABLE 3 Differentially expressed genes in human mononuclear cells in vivo (n = 12) representing the effect of rifampicin (600 mg per day for one week) vs. placebo.

Gene symbol	Description	Fold change
HBG2	Hemoglobin, gamma G	1.1
ABLIM1	Actin binding LIM protein 1 transcript variant 2	1.1
GATA2	Homo sapiens GATA binding protein 2	1.1
HBG1	Homo sapiens hemoglobin, gamma A	1.1
MOAP1	Homo sapiens modulator of apoptosis 1	1.1
LILRB2	Homo sapiens leukocyte immunoglobulin-like receptor, subfamily B (with TM and ITIM domains), member 2	-1.1
GPER	Homo sapiens G protein-coupled estrogen receptor	-1.1
IFNGR1	Homo sapiens interferon gamma receptor 1	-1.1
CISH	Cytokine inducible SH2-containing protein	-1.1
PRDM1	PR domain containing 1, with ZNF domain	-1.1
DYNLT1	Dynein, light chain, Tctex-type 1	-1.1
HSPA6	Heat shock 70kDa protein 6	-1.2
SLA	Src-like-adaptor	-1.2
KCNJ2	Potassium inwardly-rectifying channel, subfamily J, member 2	-1.2
OAZ2	Ornithine decarboxylase antizyme 2	-1.3*

*Statistically significant when false correction rate (FDR) was applied. The other transcripts are statistically significantly affected by rifampicin dosing without FDR ($P < 0.05$).

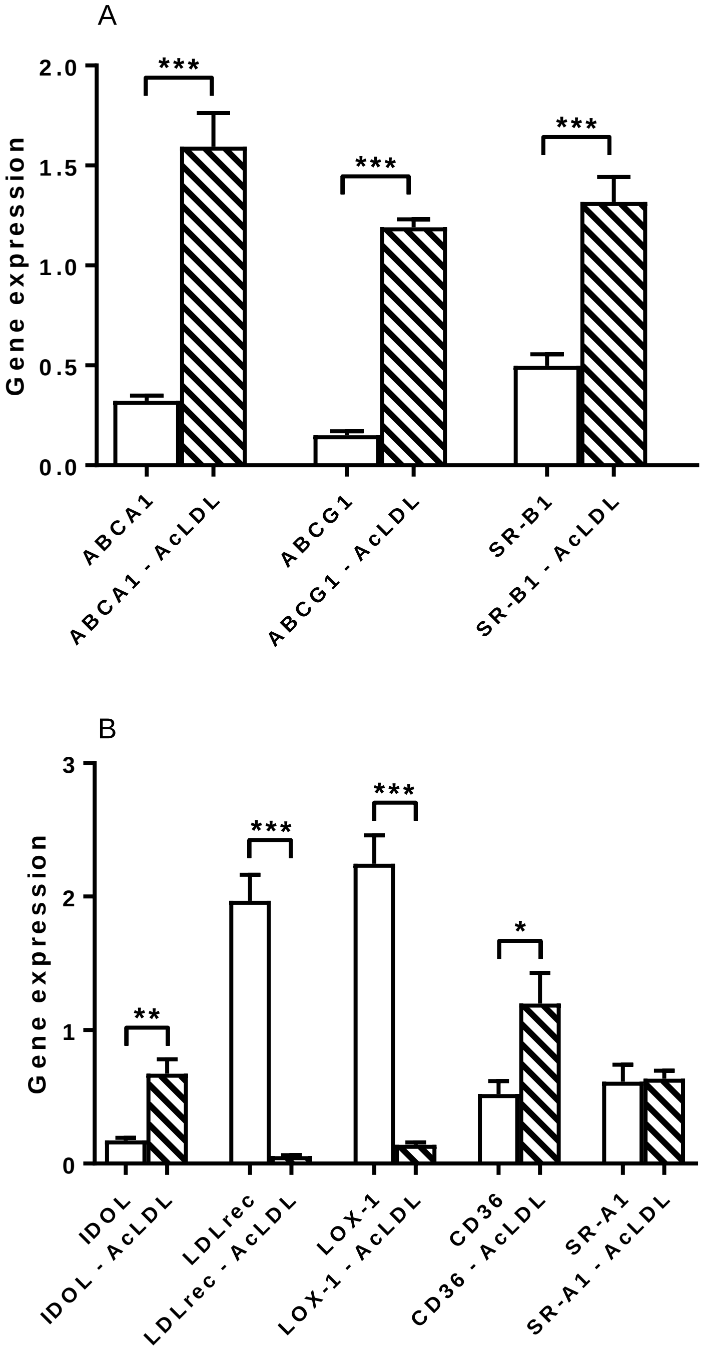

SUPPLEMENTARY FIGURE 1 The effect of acetylated LDL on the expression of cholesterol efflux transporters (A) and influx transporters and IDOL (B) in human primary monocyte-macrophages in vitro measured with quantitative RT-PCR. The blank bars show the expression without acetylated LDL treatment and the striped bars present the expression after incubation with 50 µg/ml acetylated LDL for 48 hours. The experiments were performed in four separate wells per experimental condition ($n = 4$). Data are presented as the mean + SD. *$P < 0.05$, **$P < 0.01$, and ***$P < 0.001$; unpaired two-tailed Student’s t-test.

Muscle

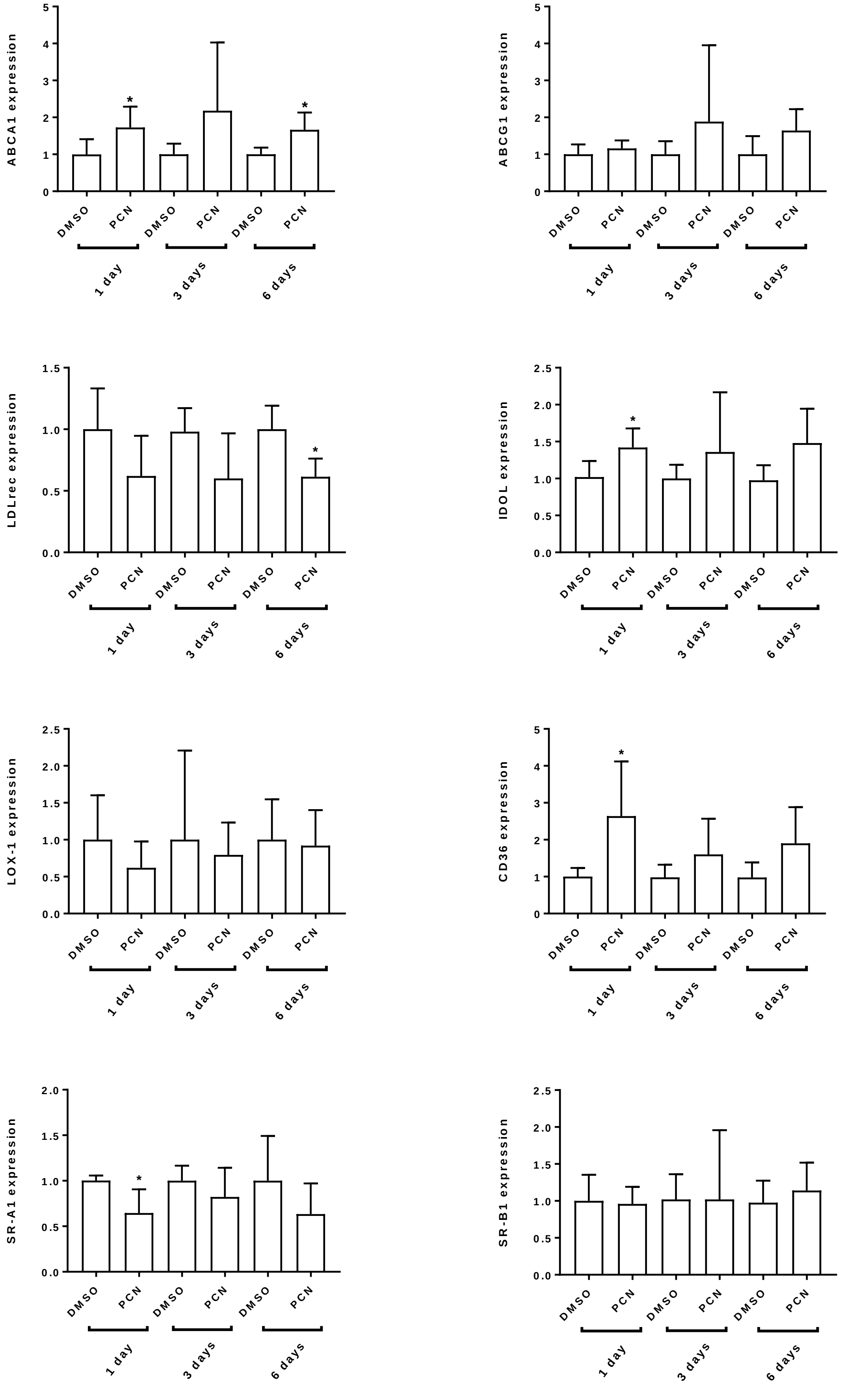
ABCA1 expression
ABCG1 expression
LDLrec expression
IDOL expression
LOX-1 expression
CD36 expression
SR-A1 expression
SR-B1 expression
DMSO
PCN
1 day
3 days
6 days
*

SUPPLEMENTARY FIGURE 2 Effect of intraperitoneal pregnenolone 16α-carbonitrile (a rat PXR agonist) 40 mg/kg versus vehicle control (DMSO) for 1, 3, or 6 days on the relative mRNA expression of cholesterol transporters in rat muscle in vivo as measured with quantitative RT-PCR. The experiments were performed with five rats per experimental condition (n = 5). Data are presented as the mean + SD. *P < 0.05 versus vehicle control; unpaired two-tailed Student's t-test.

White adipose tissue

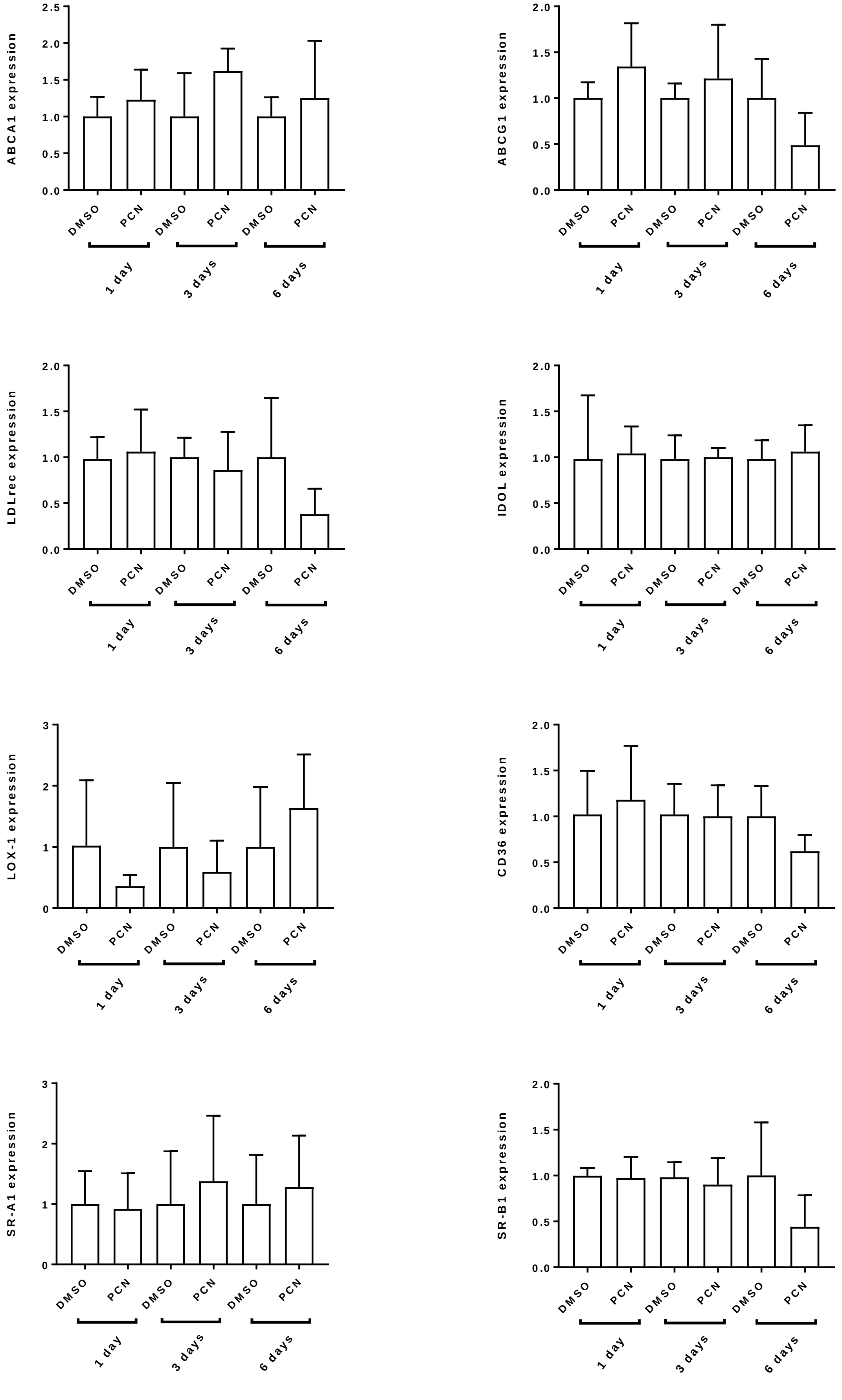

SUPPLEMENTARY FIGURE 3 Effect of intraperitoneal pregnenolone 16α-carbonitrile (a rat PXR agonist) 40 mg/kg versus vehicle control (DMSO) for 1, 3, or 6 days on the relative mRNA expression of cholesterol transporters in rat white adipose tissue in vivo as measured with quantitative RT-PCR. The experiments were performed with five rats per experimental condition (*n* = 5). Data are presented as the mean + SD.

Adrenal gland

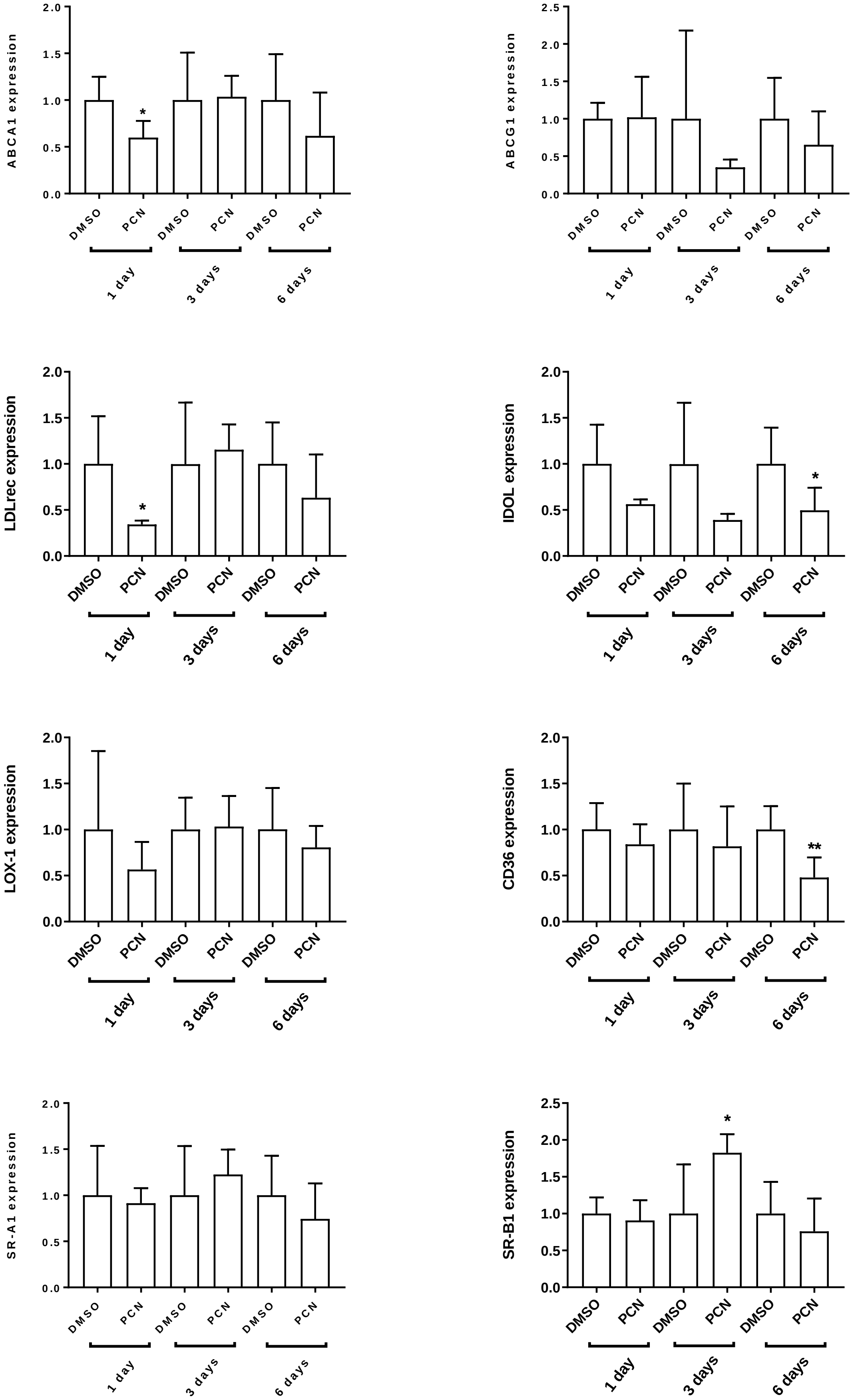

SUPPLEMENTARY FIGURE 4 Effect of intraperitoneal pregnenolone 16α-carbonitrile (a rat PXR agonist) 40 mg/kg versus vehicle control (DMSO) for 1, 3, or 6 days on the relative mRNA expression of cholesterol transporters in rat adrenal gland in vivo as measured with quantitative RT-PCR. The experiments were performed with five rats per experimental condition (n = 5). Data are presented as the mean + SD. *P < 0.05 and **P < 0.01 versus vehicle control; unpaired two-tailed Student's t-test.

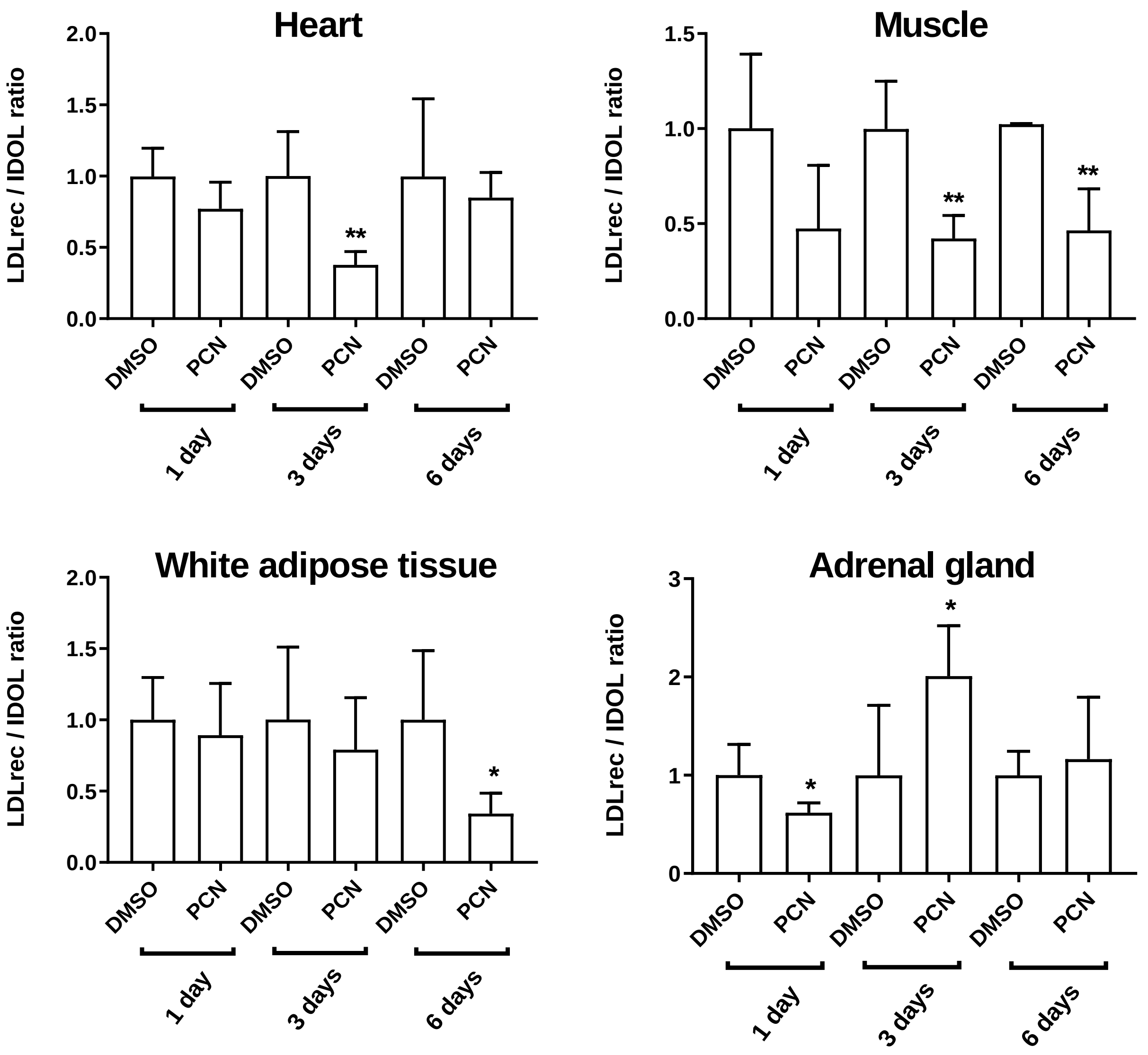

SUPPLEMENTARY FIGURE 5 Effect of intraperitoneal pregnenolone 16α-carbonitrile (a rat PXR agonist) 40 mg/kg versus vehicle control (DMSO) for 1, 3, or 6 days on the ratio of LDL receptor to IDOL expression in rat tissues *in vivo* as measured with quantitative RT-PCR. The experiments were performed with five rats per experimental condition ($n = 5$). Data are presented as the mean + SD. *$P < 0.05$ and **$P < 0.01$ versus vehicle control; unpaired two-tailed Student's t-test.

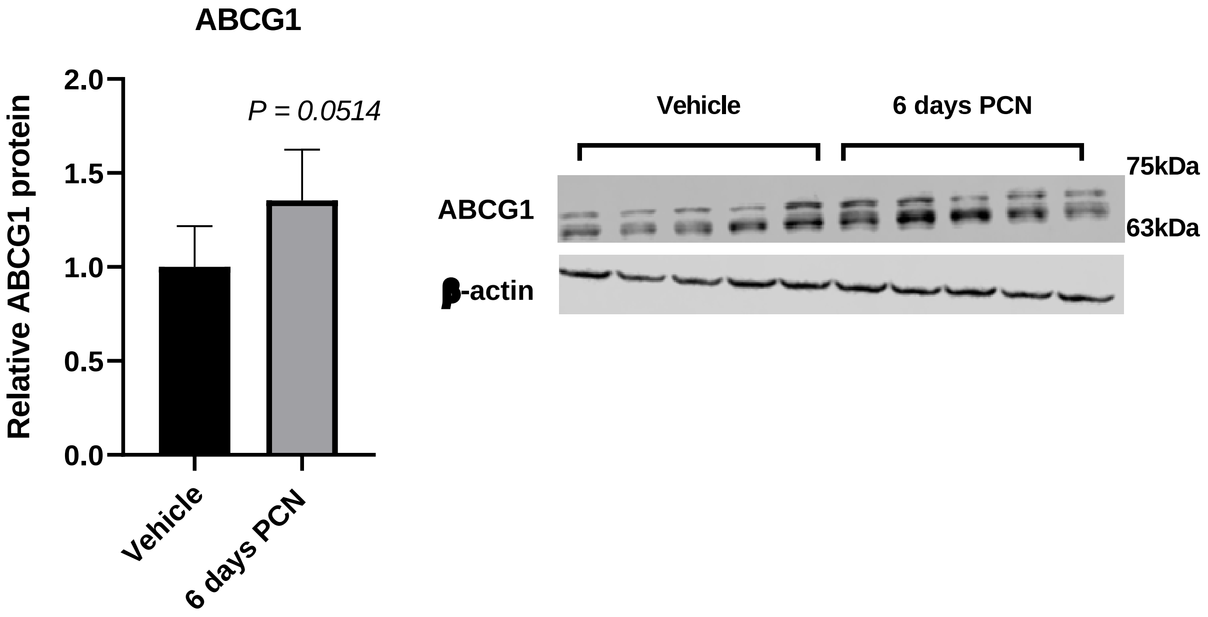

SUPPLEMENTARY FIGURE 6 Effect of intraperitoneal pregnenolone 16α-carbonitrile (a rat PXR agonist) 40 mg/kg versus vehicle control (DMSO) for 6 days on the ABCG1 protein expression in the rat heart (left ventricle). The experiments were performed with five rats per experimental condition (n = 5). Data are presented as the mean + SD. Unpaired two-tailed Student's t-test.
